# The Relation Between Emotion Understanding and Theory of Mind in Children Aged 3 to 8: The Key Role of Language

**DOI:** 10.3389/fpsyg.2018.00724

**Published:** 2018-05-15

**Authors:** Ilaria Grazzani, Veronica Ornaghi, Elisabetta Conte, Alessandro Pepe, Claudia Caprin

**Affiliations:** ^1^“Riccardo Massa” Department of Human Sciences for Education, University of Milano-Bicocca, Milan, Italy; ^2^Department of Psychology, University of Milano-Bicocca, Milan, Italy

**Keywords:** emotion understanding, theory of mind, language skills, preschool children, school-age children

## Abstract

Although a significant body of research has investigated the relationships among children’s emotion understanding (EU), theory of mind (ToM), and language abilities. As far as we know, no study to date has been conducted with a sizeable sample of both preschool and school-age children exploring the direct effect of EU on ToM when the role of language was evaluated as a potential exogenous factor in a single comprehensive model. Participants in the current study were 389 children (age range: 37–97 months, *M* = 60.79 months; *SD* = 12.66), to whom a False-Belief understanding battery, the Test of Emotion Comprehension, and the Peabody Test were administered. Children’s EU, ToM, and language ability (receptive vocabulary) were positively correlated. Furthermore, EU scores explained variability in ToM scores independently of participants’ age and gender. Finally, language was found to play a crucial role in both explaining variance in ToM scores and in mediating the relationship between EU and ToM. We discuss the theoretical and educational implications of these outcomes, particularly in relation to offering social and emotional learning programs through schools.

## Introduction

The purpose of the current work was to investigate the relationships between emotion understanding (EU), theory of mind (ToM), and language abilities (specifically receptive vocabulary) in a large sample of children with a broad age range.

In recent decades, developmental psychologists have become increasingly interested in children’s acquisition of social cognition skills, such as EU, ToM, and language (Carpendale and Lewis, 2006; Hughes and Devine, 2015). The contemporary study of children’s social cognition differs from earlier research informed by Piagetian theory, because it directly explores children’s ability to attribute epistemic – as opposed to perceptual – mental states, particularly knowledge and beliefs (Baron-Cohen et al., 1985), and non-epistemic inner states, such as emotions and affect (Harris, 1989). This line of enquiry has shown, on one hand, that children’s ability to view the self and others as possessing internal states bears key implications for their social behavior and adjustment (Garner et al., 2008; Knafo et al., 2011; Imuta et al., 2016; Longobardi et al., 2016); and on the other, that social cognition is a complex construct comprising multiple, distinct yet interrelated, abilities (Cutting and Dunn, 1999; Harwood and Farrar, 2006). Among the components of social cognition, EU, and ToM are particularly crucial to children’s ability to adjust to social life.

Language is also critically implicated in children’s gradual acquisition of social cognition abilities. Indeed, there is substantial evidence that both EU and ToM are key correlates of language competence (Milligan et al., 2007; Strand et al., 2016). However, the direction of this association has not yet been clarified. Advanced social cognition skills may be necessary to acquire language, and at the same time language may be a powerful tool for children’s acquisition of EU and ToM (Tomasello, 2003). Thus, this work aims at deepening the association among these variables.

Furthermore, the studies that have investigated the relations among all these variables have often targeted cohorts of children with a limited age range, for example focusing exclusively on preschoolers or on children undergoing their first years of formal schooling. Hence, the current study aims at investigating the relationships between EU, ToM, and language skills in a large sample of children with a broad age range, while controlling for the effects of age and gender. As necessary background, we first review existing studies that are relevant to these research aims, and afterward itemize the specific hypotheses.

### The Development of Emotion Understanding

Emotion understanding may be defined as the child’s understanding of the nature, causes, and control/regulation of emotion, or as the way in which the child identifies, predicts, and explains emotion in him/herself and others (Harris, 2008). Such definitions reflect the multi-componential nature of EU, and consequently, the complexity of the process through which children develop EU and the scope for individual variation in this area of development.

While numerous studies have examined infants’ and toddlers’ understanding of emotion (for a review see Widen and Russell, 2008), Pons et al. (2004) proposed a relatively comprehensive model tracking children’s development of EU between the ages of 3 and 10–11 years. This developmental model is borne out by empirical data from research conducted across different cultures (e.g., Albanese and Molina, 2008; Tenenbaum et al., 2008; Weimer et al., 2012; Kårstad et al., 2015). Most of these studies have used the Test of Emotion Comprehension (Pons and Harris, 2000), the instrument that was also adopted in the present study.

According to the model put forward by Pons et al. (2004), children with a typical developmental profile, independently of their gender, progress through a series of landmarks in developing nine different components of EU. At a first level (labeled *external* by the authors), children from around 3–4 years of age demonstrate mastery of three initial components: recognition of facial expressions (e.g., sadness, happiness, fear, and anger), understanding of the impact of situational factors on emotions, and the role of desires in emotion. At a second level (termed *mental*), children from around 6/7 years of age gain three further components of EU: appreciation of the role of beliefs in emotions, the impact of memory on emotions, and the distinction between outwardly expressed and privately felt emotions. At a third level (known as *reflective*), children from about 8/9 years of age are found to have acquired the three remaining components of EU: knowledge of how moral considerations affect emotions, awareness that emotions may be regulated by means of cognitive control strategies, and an appreciation of concurrent mixed feelings. Each transition from one level to another represents an increase in the child’s ability to understand the effect of internal states on emotional experience.

### The Development of an Explicit Theory of Mind

Theory of mind concerns children’s understanding of their own and others’ mental states (Carpendale and Lewis, 2006). There is evidence that children attribute intentions, emotions, desires, and beliefs from early in life, and that by taking into account such internal experiences they can predict and explain overt behaviors (Wellman, 2014).

Although very young children fail standard ToM tasks because of their limited language and executive functioning abilities, a growing body of research has revealed that they possess some knowledge of the minds of others from the beginning of the second year of life (Onishi and Baillargeon, 2005; Surian et al., 2007; Kovács et al., 2010; Yott and Poulin-Dubois, 2012). Hence, developmental psychologists have begun to distinguish between two different ToM mechanisms, which are conventionally labeled implicit and explicit (Apperly and Butterfill, 2009; Schneider et al., 2014).

Albeit with large individual differences, children’s explicit understanding of their own and others’ minds constantly develops throughout the preschool and school years, in line with a progressive set of conceptual acquisitions (Peterson et al., 2012). In this regard, Wellman (2014) has scaled explicit theory-of-mind development in early childhood, identifying a five-step progression in mind-reading abilities. Specifically, during toddlerhood and preschool years, normally developing children in Western countries successively acquire understanding of diverse desires, diverse beliefs, knowledge access, false beliefs, and hidden emotions. Indeed, mental state terms describing desires and emotions appear in their talk at about 24 months, demonstrating an ability to attribute different desires to self and others. Next, at around 3 years of age, cognitive terms related to beliefs begin to feature in children’s vocabulary, reflecting the understanding that different people can have different beliefs about the same situation. By age 4, children realize that a person may be ignorant of a piece of information that he or she does not have access to. The next important step in mind-reading, which occurs soon after at around 4-and-a-half years of age, is false-belief understanding. This attests a conceptualization of the mind as a representational system: although a given piece of information may be true, the child realizes that it is possible for someone to hold an alternative belief. The final achievement in Wellman’s scale is attained later at around 6 years and concerns awareness of hidden emotions: that is to say, appreciation of the fact that a person may feel one emotion but display another (Wellman, 2014). This last aspect of ToM is also one of the components listed by Pons et al. (2004) in their proposed model of EU development.

Among the steps involved in acquiring a ToM, false belief understanding has been particularly extensively investigated, because it represents a key developmental milestone. Indeed, success on false-belief tasks requires the insight that internal states can potentially be at odds with external reality in everyday life situations (Hughes and Devine, 2015). As noted above, at around 4 years, children start to acquire the explicit understanding that people may have erroneous beliefs about reality, with consequences for their behavior. However, this form of recursive mentalistic reasoning has not fully developed by the end of the preschool years: only at 6 or 7 years do further cognitive gains enable children to acquire second-order false-belief understanding. Thus, around the start of the primary school period, children begin to develop the awareness that a person can hold beliefs about others’ beliefs, thereby laying the ground for new forms of reasoning and behaviors (Miller, 2012).

### Emotion Understanding and Theory of Mind

Evidence of a relationship between EU and ToM comes from a significant body of research, most of which has been conducted with preschoolers. In a pioneering study, Cutting and Dunn (1999) investigated social understanding in a sample of over 100 preschool children aged 3 and 4 years old. While they found a correlation between ToM and EU, their regression analyses suggested that variance in ToM did not contribute independently of other variables (e.g., age) to explaining variance in EU, or vice versa. The authors therefore speculated that, at least at around 4 years, these two domains of social cognition remain distinct and that the correlations they had found were due to moderating factors such as age, linguistic competence, or family background (e.g., social class, size of family, family usage of internal state lexicon, etc.).

Another cross-sectional study that examined the associations between EU and ToM in preschoolers was carried out by Harwood and Farrar (2006). These authors focused on the connection between affective perspective-taking and ToM in a sample of 42 children aged between 3 and 5 years, reporting significant positive relationships between EU and ToM, independently of age and receptive language skills.

More recently, Weimer et al. (2012) conducted a study with 4-and-a-half to 6-and-a-half year olds (*n* = 78), using similar instruments to those adopted in the current research. They too found a correlation between understanding of the external causes of emotion and ToM. Similar findings emerged from a study carried out by Ornaghi et al. (2016) in the same age range as the participants in Weimer and colleagues’ research. Focusing on how language and false-belief understanding mediate the influence of emotion comprehension on prosocial orientation, the authors reported positive significant correlations between EU and ToM, even when the effects of age and gender had been controlled for.

Compared to earlier studies, Bender et al. (2011) recruited a slightly older sample of children, specifically aged between 5 and 7 years (*n* = 45), to examine the relationship between ToM and EU competencies, in the domain of belief-based emotions in self and others. These authors too found positive correlations between the two domains.

Interesting data (also in relation to the purposes of the present work) on both the association between EU and ToM and the direction of influence between the two variables has been provided by longitudinal studies. For example, O’Brien et al. (2011) assessed the association between knowledge of emotions and ToM in a large sample (*n* = 507) of preschoolers over the period between 3 and 4 years. Hierarchical multiple regression analyses suggested that understanding of emotion develops early and informs children’s comprehension of others’ thinking. Specifically, EU at age 3 predicted variance in performance on a ToM task at age 4. Evidence that EU influences ToM skills has also come from a recent longitudinal study conducted by Kuhnert et al. (2017) with a sample of older children. The authors analyzed, among other variables, the relationship between ToM and EU at 5 and 7 years old, reporting strong significant associations between EU at 5 years of age and ToM skills 2 years later. Again, this finding suggests that children’s EU precedes and influences their acquisition of ToM skills.

### Language Ability and Children’s Social Cognition: a Complex Relationship

Evidence that language plays a crucial role in false-belief understanding has come from both cross-sectional (de Villiers and de Villiers, 2000; Astington and Baird, 2005; Longobardi et al., 2016) and training studies (Hale and Tager-Flusberg, 2003; Lohmann and Tomasello, 2003; Sprung et al., 2015). Although the direction of influence is still debated (Milligan et al., 2007), a substantial body of research findings suggests that the relationship between ToM and language may be bidirectional (Slade and Ruffman, 2005). However, the effect of language on ToM seems to be stronger than the other way around, and language plays a crucial role in fostering children’s understanding of the mind. Furthermore, different aspects of language (semantic, syntactic, and pragmatic) all seem to be related to ToM. In a meta-analysis of the relationship between language ability and false-belief understanding, Milligan et al. (2007) showed that ToM tasks demand comprehension of both mental state terms and complex syntax in children. A strong positive relationship between language competence and advanced ToM abilities has also been reported in primary school children (Astington and Pelletier, 2005; Grazzani and Ornaghi, 2012).

In intervention programs based on a conversational approach, children who took part in targeted language activities displayed greater gains in performance on ToM tasks than children in a control group who received no linguistic training. For example, San Juan and Astington (2017) have recently reported that preschoolers’ performance on false-belief tasks significantly improves after they receive training in epistemic verbs. Similarly, encouraging preschoolers and school-age children to use emotion-state terms while discussing their own and others’ emotional experiences results in more advanced levels of EU (Tenenbaum et al., 2008; Grazzani and Ornaghi, 2011; Ornaghi et al., 2015). Conversational activity facilitates the transformation of children’s implicit knowledge into explicit awareness of their own and others’ emotional states, leading to gains in their understanding of emotional experience, including at a very early age, as recently reported by Grazzani et al. (2016a,b) and Ornaghi et al. (2017).

Finally, further evidence of the role played by language in the development of children’s social cognition comes from studies investigating ToM performance in children with specific language impairments and delays. These children display significantly poorer social cognitive knowledge and emotion comprehension than their typically developing peers (Marton et al., 2005; Spackman et al., 2006; Nelson et al., 2011; Rieffe and Wiefferink, 2017). Evidence of a causal link between language and ToM is provided by studies with deaf children whose language development and false-belief understanding are both delayed (Slaughter and Peterson, 2011).

### The Role of Gender in Children’s Developing Social Cognition

The effects of gender have been assessed in some but not all of the studies investigating children’s social cognition. In general, when gender has been controlled for, it has been found to make little (e.g., Kårstad et al., 2015) or no contribution (e.g., Pons et al., 2002, 2004; Pons and Harris, 2005; Grazzani et al., 2015; Ornaghi et al., 2016) to explaining variance in EU. In a recent study with a sample of 3- to 8-year-old children, Fidalgo et al. (2017) found that EU was unaffected by gender in relation to eight out of the nine components of emotion understanding assessed by the TEC.

With regard to gender and ToM, in general girls have been found to perform slightly better than boys on false-belief tasks (Charman et al., 2002; Slaughter et al., 2015). It has been argued that this is because females are more proficient in mental state talk (Milligan et al., 2007).

### The Present Study

As discussed above, EU and ToM are two constructs that have been the object of in-depth research in recent years, in relation to their association with one another and to how they may both be connected with language abilities. Given that EU, ToM, and language ability all develop gradually over the period spanning the preschool and primary school years (e.g., Moran, 2013), it is of interest to establish how the relations among them vary as a function of age. As far as we know, no studies to date have examined these variables and their interrelationships in a large sample of children with a broad age range. Hence, in an attempt to fill this gap in the literature, this study was designed to investigate the relationships among EU, ToM, and language skills in a sizeable sample of children aged between 3 and 8 years, while controlling for the effects of age and gender.

More specifically, and in line with the most recent research findings reviewed above, we hypothesized (H1) that all the different levels of EU (external, mental, and reflective) would be positively correlated with ToM ability. Moreover, on the basis of O’Brien et al. (2011) and Weimer et al.’s (2012) findings, we expected (H2) that children’s EU scores would explain variance in their ToM scores, independently of age and gender. Finally, we expected that (H3) language ability would be associated with ToM, significantly contributing to explaining variance in its performance. In addition, in order to further deepen the relationship among these variables, the present study also explored the direct effect of EU on ToM when the role of language was evaluated as a potential exogenous variable in a single comprehensive model.

## Materials and Methods

### Participants

The sample comprised 389 children (194 girls), whose ages ranged from 37 to 97 months (3–8 years of age; *M* = 60.79 months; *SD* = 12.66). They were almost equally divided according to the different age group. All participants were typically developing children with no psychological, behavioral, or language problems, who were recruited through kindergartens and primary schools in Northern Italy. They were all native Italian speakers and came predominantly from middle-class socioeconomic backgrounds, given that the majority of their parents held a high school diploma or university degree (79%) and were either in white-collar employment or self-employed professionals (72%).

### Ethics Statement

The study was conducted in conformity with the recommendations of the University of Milano-Bicocca Ethics Committee. Parental written informed consent was obtained for all participants in keeping with the Declaration of Helsinki.

### Measures

All children were tested individually in a quiet room at their preschool or primary school. A series of standardized instruments designed to assess the variables under study were administered in counterbalanced order over two sessions held on different days. The instruments were selected on the basis that they are validated measures, and particularly suited to the age of the participants.

#### Test of Emotion Comprehension (TEC, Pons and Harris, 2000)

In the current study, we used the standardized Italian version developed by Albanese and Molina (2008). This test has the merit of concurrently evaluating numerous aspects of EU across a large span of age. More precisely, it evaluates 3- to 11-year-old children’s comprehension of the nature, causes, and regulation of emotion, as well as their current stage of EU development in relation to the external, mental, and reflective levels described above (Pons et al., 2004). The test materials consist of a picture book with nine simple cartoon scenarios corresponding to the nine components of EU. Beneath each scenario, there are four emotional outcomes represented as facial expressions. The examiner reads a brief statement (e.g., This child has just received a present for his birthday) or story describing the scenario and then shows the child the four faces representing four different emotional states. The child is required to indicate which of these faces best matches the emotion experienced by the story character. Children receive a score of 1 for each correct answer, thus obtaining total scores ranging between 0 and 9. The composite reliability coefficient (Raykov, 1997) for the TEC was CR = 0.734.

#### False-Belief Understanding Battery

Preschool children were assessed via two first-order false belief tasks, namely a false-belief location change task (Baron-Cohen et al., 1985) and an unexpected content task (Perner et al., 1987). In the first task, children were asked to predict where a story character would look for an object that had been moved from one place to another without his/her knowledge, and were assigned a score of 1 for the correct answer and 0 for a wrong answer. In the second task, children were first asked what they believed to be the contents of a box that looked as though it held sweets; after they had guessed, they were shown that the box in fact contained pencils. They were then asked what they thought another person, who had not been shown the true contents of the box, would think was inside it. Participants were assigned a score of 1 for the correct answer and 0 for a wrong answer. Scores for the battery were summed, yielding a possible maximum total score of 2. Primary school children, during the second half of their school year, were assessed using two second-order false belief prediction tasks, namely the look-prediction task and the say-prediction task (Astington et al., 2002; Liverta Sempio et al., 2005). In the look-prediction test the child is asked to predict where the protagonist of a story thinks that another story character will look for an object; in the say-prediction test the child is asked to predict what the protagonist thinks another character will say about a gift he will receive for his birthday. For each of the two tasks, children were asked two control questions to ensure that they had understood the story, a first-order question and a second-order question, and finally invited to justify their response to the latter. The two false belief tasks were administered in counterbalanced order. A conservative scoring criterion was adopted for both tasks: a score of 1 was awarded only if children both provided a correct response to the second-order question and justified their answer appropriately, with a score of 0 being awarded in all other cases. Again, an aggregate score for the false belief battery was calculated for each participant by summing the scores for the two individual tasks, yielding a maximum possible score of 2. The composite reliability coefficient (Raykov, 1997) for the ToM battery was CR = 0.786.

#### Peabody Picture Vocabulary Test (PPVT, Dunn and Dunn, 1981)

The Italian standardized version of the test (Stella et al., 2000) was used. This instrument evaluates the receptive vocabulary of children between 3 and 12 years and consists of 180 cards, each containing four numbered illustrations among which the child is asked to indicate the one that corresponds to the word called out by the examiner. Scoring was carried out following the standard procedure, with 1 point assigned for each correct answer and 0 for each wrong answer.

## Results

The data analysis was conducted in two stages. First, assumptions for multivariate analysis (i.e., skewness and kurtosis) were checked and the data assessed for missing values and both uni- and multivariate outliers. The range ±2 was adopted as the threshold for viewing a set of scores as normally distributed (George and Mallery, 2010). Mahalanobis distances were then computed, with the *p-*value set at 0.001. The datasets for all scales were found to be normally distributed. Eight participants (2%) were identified as presenting outlying values and were therefore omitted from the subsequent analyses. Missing values were handled via a listwise deletion method (leading to three additional participants being skipped).

**Table 1** presents the main descriptive statistics, as well as the zero-order correlations found among the study variables. Mean scores and standard deviations of all variables as a function of children’s groups of age are available in **Table 2**.

**Table 1:** Means, standard deviations, skewness, and zero-order correlations for the investigated variables.

	1	2	3	4	5	6	7
Gender (1)	-						
Age (in months) (2)	0.01	-					
ToM battery (3)	0.03	0.44**	-				
EU-external (4)	0.02	0.46**	0.34**	-			
EU-mental (5)	-0.05	0.52**	0.30**	0.35**	-		
EU-reflective (6)	-0.01	0.42**	0.30**	0.32**	0.27**	-	
Language (7)	0.03	0.73**	0.50**	0.59**	0.51**	0.43**	-
Mean	-	60.8	1.15	2.23	1.43	0.96	64.66
Standard deviation	-	12.7	0.80	0.94	0.96	0.83	28.14
Skewness	-	0.62	-0.27	-0.94	0.10	0.47	0.16


*Gender was coded as a dummy variable (0 = boys; 1 = girls). ^∗∗^*p* < 0.001.*

**Table 2:** Descriptive statistics by children’s age group (*N* = 389).

	Age in months									
	37–48		49–60		61–72		73–84		85–97	
	m	ds	m	ds	m	ds	m	ds	m	ds
Emotion understanding (EU)	2.56	1.55	4.19	1.66	5.16	1.60	6.20	1.41	7.14	1.01
Theory of mind (ToM)	0.43	0.66	1.10	0.076	1.31	0.65	1.60	0.50	1.79	0.41
Language ability (vocabulary)	31.3	17.1	54.6	19.5	74.4	22.7	91.1	17.1	112.7	9.65


In general, the correlational analysis revealed a set of statistically significant, moderate (*r* ≃ 0.40) and positive relationships among variables. The most robust correlation was found between age and language ability (vocabulary) (*r* = 0.73, *p* < 0.001). Furthermore, language was positively correlated with ToM ability (*r* = 0.50, *p* < 0.001) and with the three dimensions of EU (*r_MEAN_* = 0.51, *p* < 0.001). On the contrary, gender was not correlated with any of the other variables.

To investigate whether children’s EU scores explained the variance in ToM scores, regression analysis was carried out. The regression model was designed to assess variance in ToM scores across three consecutive steps. At the first step (1), the role of age and gender was evaluated. Next (Step 2), the influence of the three dimensions of EU (external, mental, and reflective) was explored by entering them in the regression model. Finally, at Step (3), the effect of language was assessed. Multicollinearity issues were ruled out given that the average variance inflation factor was 0.699 indicating low collinearity. The regression model was assessed after each step in terms of statistically significant variations in the coefficient of determination (*R^2^*) and standardized beta weights (β). The independent variables were centered prior to entry in order to enhance the comparability of the beta weights (Aiken and West, 1991; Echambadi and Hess, 2007). Cohen’s (1992) effect size was computed for the model.

Children’s age and gender were entered together at Step 1. The model was statistically significant, *F*(2,386) = 45.44, *p* < 0.001, and accounted for approximately 19% of explained variance. Only the variable age explained variability in ToM scores (β = 0.44, *p* < 0.001). At Step 2, *F*(5,383) = 22.91, *p* < 0.001, the inclusion of the three measures of emotion understanding led to a statistically significant increase in explained variance (*R^2^*= 0.23). Specifically, the external (β = 0.15, *p* < 0.005) and reflective (β = 0.12, *p* < 0.05) dimensions of EU were found to have a significant impact on ToM scores. Finally, when language was entered into the regression model, *F*(6,382) = 23,36, *p* < 0.001, explained variance increased by 4% (*R^2^*= 0.27). The beta weight for language ability (β = 0.32, *p* < 0.001) implied that this variable should be considered a robust determinant of ToM scores (see **Table 3**). It is interesting to note that when the model accounted for the effect of language ability, both the external and reflective components of EU ceased to have a statistically significant association with ToM scores. The model reported a *medium* total effect size (f^2^ = 0.35) and provided support to both H2 and H3. This result suggests that language ability plays a crucial role in the relation between EU and ToM, setting up the conditions for which the components of EU favor the development of ToM.

**Table 3:** Regression outcomes (standardized *beta weights*) for target variable ToM (*N* = 389).

		β	*SE*	*t*	*p*
Step 1					
	Gender	0.025	0.073	0.552	0.581
	Age	0.436^∗∗^	0.003	9.51	0.000
Step 2					
	Gender	0.029	0.072	0.646	0.518
	Age	0.281^∗∗^	0.004	4.82	0.000
	EU-external	0.149^∗∗^	0.044	2.88	0.004
	EU-mental	0.068	0.044	1.29	0.199
	EU-reflective	0.117^∗^	0.050	2.34	0.020
Step 3					
	Gender	0.020	0.070	0.454	0.650
	Age	0.123^†^	0.004	1.84	0.067
	EU-external	0.056	0.046	1.02	0.307
	EU-mental	0.025	0.044	0.465	0.642
	EU-reflective	0.086^†^	0.048	1.73	0.083
	Language	0.325^∗∗^	0.002	4.46	0.000


*^†^*p* < 0.10, ^∗^*p* < 0.05, ^∗∗^*p* < 0.005.*

In order to further evaluate the association between EU and ToM performance when the effect of language was partialled out, *exploratory mediation analysis* was performed (Cohen et al., 2007; MacKinnon, 2008). Precisely, we were interested in evaluating whether and to what extent language ability (M) accounted for the relationship between EU (X) and ToM (Y). The model was tested by using age (C1) and gender (C2) as confounding variables that might be associated to other constructs and that “might falsely accentuate or obscure” their associations (Meinert, 1986, p. 285). The idea of full mediation should be accepted if, once controlled for the effect of the mediators, the association (c′) between the criterion and the determinant variables is no longer statistically significant (Baron and Kenny, 1986).

Mediation analysis was conceptually developed from a confirmatory perspective (Baron and Kenny, 1986); however, the available theory on certain emerging fields of knowledge might be still limited and, as a result, specific hypotheses about associations among variables might not be defined *a priori*. Under these particular circumstances, the adoption of techniques for exploratory data analysis can be of paramount importance, especially in the context of mediation analysis (MacKinnon, 2008).

We agreed that, in using such procedures, the likelihood of Type I errors increases (Behrens et al., 2013) along with the chances of highlight statistically significant mediators. Thus, to apply “some control over family-wise error rates” (Sarfaraz et al., 2017, p. 736), we decided to adopt Bonferroni correction. The cut-off point for statistical significance was set to be equal to 0.01. Mediation test was conducted by using Hayes and Preacher’s (2010) macro for SPSS 22. The macro provided estimates of effects by dividing the total effect of X on Y into two parts: the direct effect of X on Y, and the indirect effect of X on Y through a mediator M (Hayes, 2009). In addition, bootstrap non-parametric resampling procedure with 5,000 bootstrap sample simulations was applied (for details see Shrout and Bolger, 2002). Estimates of the indirect effect along with their 95% confidence intervals were provided.

**FIGURE 1:**
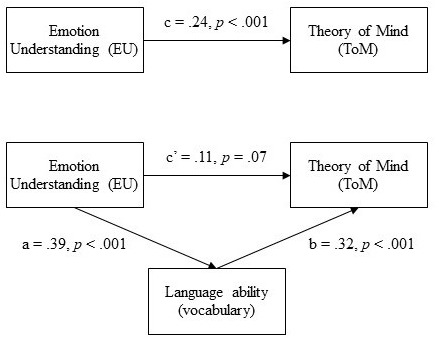
The model of mediation with total (c) and direct effect (c′) of EU on ToM. Standardized effects were reported.

Results of mediation analyses (see **Figure 1**) revealed that the total standardized direct effect of EU on ToM, without considering the effect of language ability, was medium-small in size (*β*_X,Y_ = 0.24) and statistically significant (*p* < 0.001). The analysis of the association between language (vocabulary) and ToM suggested a positive, standardized medium (*β*_M,Y_ = 0.32) and statistically significant (*p* < 0.001) effect. Finally, the regression model indicated that the standardized direct effect (*β*_X,YjM_) of EU on ToM, considering the effect of language ability, was equal to 0.11 and represented a not statistically significant effect (*p* = 0.069). Bootstrap analysis of the indirect effect of vocabulary on the association between EU and ToM suggested a bias corrected 95% confidence interval that did not include the zero value, CI [0.07, 0.18]. Analysis of the covariates revealed that neither gender (*t* = 0.48, *p* = 0.62) nor age (*t* = 1.81, *p* = 0.07) played a statistically significant role.

These results, along with the statistical significance of the remaining paths, supported the idea of a full mediation model accounting for 27% of the variance in ToM performance [*R*^2^ = 0.27; *F*(4,384) = 34.75, *p* < 0.001].

## Discussion

The aim of the current study was to further advance our understanding of the relationship between children’s social cognition and language competence by assessing these abilities in a sample of 3- to 8-year-old children. Although this topic has been extensively investigated in recent years, the novelty and strength of the present work is that it was conducted with a wider age range than customary, involving both preschool and primary school children across a 5-year span of ages. In relation to our research hypotheses, we obtained three main findings which we now discuss below, including in relation to their possible applications. First, a statistically significant positive correlation was found between children’s EU and ToM across the entire age range under study. Second, ToM scores were accounted for by children’s EU, particularly the external and reflective dimensions. Finally, language ability was not only significantly related to both EU and ToM but also played a crucial role in explaining differences in ToM performance, independently of age and gender. In fact, results of exploratory mediation analyses supported the idea that language fully mediated the relationship between EU and ToM scores.

With respect to the first outcome, our results are in keeping those of earlier studies that had already identified positive associations between EU and ToM in both preschool and school samples (Harwood and Farrar, 2006; Bender et al., 2011; Weimer et al., 2012; Ornaghi et al., 2016). Given that our own study was conducted with both preschoolers and primary school children, we were able to monitor these associations across different age groups, and to confirm that the relationship between the two domains of social cognition is preserved as the child progresses from first-level to second-level forms of representation and meta-representation (Wellman, 2014). During the primary school years, children’s emotion understanding comes to include complex competences such as the awareness that people’s emotion displays do not always match their current inner emotional experience, or that it is possible to simultaneously experience emotions of both negative and positive valence, or that people’s moral values can influence their emotional experience (Pons et al., 2004). The last-mentioned abilities require greater capacity to engage in abstract thinking (Harris, 2008). In parallel, ToM is also developing further: from 6/7 years onwards, not only are children able to represent the mind of another person with its false beliefs, but they also begin to engage in recursive thinking, in which one meta-representation is contained in another (“I think that you think that he thinks ‘X”’), a skill that is required to succeed on second-order ToM tasks (Miller, 2012; Wellman, 2014).

Based on the literature reviewed above, we predicted that children’s EU would explain variance in their ToM ability, independently of age and gender. The outcomes of the regression analysis confirmed this hypothesis, given that both the external (typical of preschool children) and reflective (typical of school-aged children) components of EU contributed to explaining variability in children’s ToM performance. This last-mentioned outcome is in line with cross-sectional and longitudinal data obtained in studies of English-speaking preschoolers, such as the work of O’Brien et al. (2011), who found that early EU predicts later ToM (and not the reverse), and that of Weimer et al. (2012), who concluded that multiple components of EU (notably, those comprising the ‘external’ level) wield an effect on ToM ability.

With regard to the third finding, language was significantly associated with both EU and ToM, in line with the outcomes of numerous existing studies and meta-analyses which have found that children’s language ability is related to their false-belief understanding, at least up to age 7 years (Milligan et al., 2007). In addition, the regression analysis showed that language ability contributed to explaining ToM performance, accounting for an additional 4% of variance with respect to EU. Interestingly, the influence of language on ToM scores remained significant even after the effect of age had been controlled for. This means that across the 5-year span under study, children’s language significantly affects their ToM skills, particularly the receptive vocabulary that fully mediated the relationship between EU and ToM. There is abundant cross-sectional and longitudinal evidence of a relationship between language and ToM, for example in relation to the effect of adults’ mental-state language or the impact of child-adult conversation on children’s inner states (Milligan et al., 2007; Aznar and Tenenbaum, 2013). Nevertheless, these outcomes have primarily been obtained with children aged between 2 and 5/6 years, while fewer studies have been conducted with school-aged children (Grazzani and Ornaghi, 2012). Thus, our findings -given the overall robustness of the effect size obtained- support the hypothesis that during the primary school years, language continues to represent a valuable tool for executing explicit ToM tasks entailing complex representations with double embeddings, e.g., “she thinks that he thinks that….” Success on second-order false belief tasks reflects gains in metacognitive ability that are still ongoing at 8 years and beyond (Grazzani Gavazzi et al., 2011), as teaching-learning processes grow in complexity and children become increasingly proficient at using language to reflect on thinking (Astington and Pelletier, 2005; Grazzani and Ornaghi, 2012).

Although this study contributes to the existing literature on the links between age, EU, language, and ToM, its key limitation is that, as a cross-sectional study, it does not allow us to confirm causal relations among these variables.

### Educational Implications

The current findings suggest that language is a crucial factor in children’s developing social cognition, including their EU and particularly their ToM, across different ages. Consequently, both preschools and primary schools should offer educational activities and games that provide children with the opportunity to *use language*, both oral and written, to discuss emotional-affective and cognitive inner states.

In actual fact, recent years have seen an increase in language-based intervention at school designed to foster children’s EU and ToM development. Such educational programs have mainly involved book-reading and conversations about story characters’ inner states, and their usefulness has been borne out by the outcomes of numerous training studies (e.g., Ornaghi et al., 2011; Aram et al., 2013).

However, it is crucial that similar opportunities be provided to primary school children too. Indeed, the development of explicit mindreading continues beyond the preschool years and is influenced by cultural tools, first and foremost by the different components of language competence (Heyes and Frith, 2014). On one hand, conversations about the mind have been found to foster gains in school-aged children’s mental-state reasoning (e.g., Lecce et al., 2014; Mori and Cigala, 2016). On the other hand, from the outset of their primary education, children are required to produce increasingly complex and diversified narratives describing their own and others’ internal states; yet, they are often found to use a limited number of mental state words in their written stories, implying that primary school teachers can help to overcome this deficiency (Longobardi et al., 2014) by encouraging their students to make appropriate use of emotional and cognitive terms.

In summary, the present data are consistent with the claim that language maintains a fundamental role in social cognition development during the transition from preschool to school years. These data add new evidence, through exploratory mediation analysis, about the crucial role of language ability, more precisely the receptive vocabulary. According to our findings, this ability fully mediates the relation between EU and ToM, accounting for substantial variance in preschool and primary school children’s ToM.

## Author Contributions

IG made a leading contribution to designing the research, interpreting and discussing the data, and drafting the manuscript. VO made a key contribution to designing the research, collecting and coding the data, and drafting the manuscript. AP made a key contribution to analyzing and interpreting the data. EC made a key contribution to reviewing the literature, collecting the data, and drafting the manuscript. CC contributed in collecting the data.

## Conflict of Interest Statement

The authors declare that the research was conducted in the absence of any commercial or financial relationships that could be construed as a potential conflict of interest.
